# Differential impact of resilience on demoralization and depression in Parkinson disease

**DOI:** 10.3389/fpsyt.2023.1207019

**Published:** 2023-07-24

**Authors:** John M. de Figueiredo, Boheng Zhu, Amar S. Patel, Robert Kohn, Brian B. Koo, Elan D. Louis

**Affiliations:** ^1^Department of Psychiatry, Yale University School of Medicine, New Haven, CT, United States; ^2^Department of Neurology, Yale University School of Medicine, New Haven, CT, United States; ^3^Department of Psychiatry, Brown University School of Medicine, Providence, RI, United States

**Keywords:** resilience, demoralization, depression, Parkinson, neuropsychiatry

## Abstract

**Objectives:**

The objective of this study was to study the interrelations of demoralization, depression, and resilience in patients with Parkinson disease, and, more specifically, to determine if higher resilience in patients with Parkinson disease is associated with lower demoralization, lower depression, or both.

**Methods:**

Outpatients with Parkinson disease (*N* = 95) were assessed for demoralization, depression, and resilience, as well as sociodemographic, clinical, and treatment-related variables. Bivariable associations, standard regressions, linear regression with copula correction, and correspondence analysis were used to analyze the data.

**Results:**

Although the bivariable association between resilience and depression was statistically significant, the association ceased to be significant when demoralization was taken into consideration in both standard regressions and linear regression with copula correction. By contrast, the association between resilience and demoralization was significant when depression was not taken into consideration and continued to be significant when depression was taken into consideration. Correspondence analysis revealed that low resilience was strongly related to demoralization combined with depression, whereas normal resilience was closely correlated with depression without demoralization.

**Conclusion:**

These results expand our understanding of resilience by suggesting that it is a mechanism evolved to reduce or prevent demoralization and not just depression. Reducing demoralization and strengthening resilience as part of a comprehensive treatment plan are likely to improve the prognosis of Parkinson disease.

## Introduction

With a prevalence of 1–2% and an incidence of 108 to 212 per 100,000 person-years, Parkinson disease (PD) is one of the most common neurodegenerative diseases in the United States and worldwide (1, 2). Progressive loss of nigrostriatal dopaminergic neurons in the substantia nigra pars compacta and pathology in other brain regions results in both motor signs (bradykinesia, rigidity, resting tremor) and non-motor manifestations (e.g., depression, hallucinations, cognitive impairment, hyposmia, sleep disorders, autonomic dysfunction) (3).

Recently attention was drawn to demoralization in patients with PD (4–7). Over the past few decades, demoralization, a treatable condition, has emerged as a key concept in both psychiatric and non-psychiatric medical literature (8–13). Demoralization is characterized by expressions of distress, such as inability to cope, sense of failure, and loss of purpose and meaning, together with a feeling of entrapment (subjective incompetence), sometimes progressing to helplessness, hopelessness, existential despair, demands for euthanasia, and desire for suicide or death (12, 13). Demoralization has been found in patients with a variety of diagnoses, such as cardiac illness, cancer, essential hypertension, endocrine diseases, primary aldosteronism, and syncope as well as schizophrenia and major depressive disorder or in victims of predicaments, such as refugees, with prevalence ranging from 13 to 52%, partly due to population and methodological differences (8–10). Patients who are isolated or jobless, have less perceived social support, or have poorly controlled physical symptoms, or inadequately treated anxiety and depressive disorders are at increased risk for demoralization (8). Findings on marital status are contradictory. Most studies found that people who are single are at higher risk for demoralization (8), while a study of palliative care patients in Hong Kong found higher risk among those who were married (14).

Depression and demoralization have different presentations and trajectories and require different interventions. While they may occur together, their overlap is relatively modest (8–10). In depression, anhedonia and anergia may be present, and the magnitude of motivation is lacking even when the course of action is known. In demoralization, anhedonia and anergia are absent and a willingness to overcome the predicament is hampered by uncertainty about the course of action to be taken (direction of motivation or cognitive map) (15).

The distinction between demoralization and depression has important implications for understanding the diagnosis, pathophysiology, treatment, and prognosis of both conditions. This is suggested by the findings that hopelessness (an advanced stage of demoralization) predicts suicidal ideation better than depression in both cancer patients (after controlling for mental disorders) and psychiatric patients, and in major depressive disorder, hopelessness predicts non-response to antidepressant treatment (16–18).

Distinguishing depression from demoralization is particularly important in PD because, as shown by our previous studies, demoralization has a prevalence of 18.1%; lifetime histories of both depression and demoralization are more likely in patients than controls; demoralization explains disruptions in the quality of life better than depression; and demoralized patients are more likely than controls to have suicidal ideation (4–7). It is important to note that suicide is more common in PD patients than in the general population after controlling for mental disorders (19).

Success in the adaptation to the consequences of neurodegenerative diseases is largely propelled by the patient’s resilience. Several definitions of resilience have been proposed (20, 21). As intended here, resilience refers to the capacity to withstand, overcome, and recover or bounce back from a specific perceived stress (in this case, PD) or minimize that perceived stress in the long run. This future orientation of resilience hypothetically requires the construction of a cognitive map to guide the recovery from, or minimization of perceived stress (22). Resilience is the result of a dynamic biopsychosocial process and potentially the polar opposite of subjective incompetence (sometimes progressing to helplessness and hopelessness), the clinical hallmark of demoralization (7, 12).

Research by our team on the same group of patients found that in PD, demoralization is highly associated with depression, but not completely; lifetime histories of both depression and demoralization are more likely in patients than in controls; demoralization explains disruptions in health-related quality of life better than depression; demoralized patients are more likely than controls to have suicidal ideation; and that depression, anxiety, and subjective incompetence are mediators between perceived stress and demoralization, with subjective incompetence being the largest contributor to demoralization, and depression connected to demoralization indirectly *via* subjective incompetence (4–7).

A handful of studies have examined the relationship between resilience and depression in PD. A study of 83 PD patients found resilience associated with optimism, quality of life, and less apathy, depression, and fatigue (23). A cross-sectional survey of 138 adults with PD found resilience associated with less depression, less apathy, and greater life satisfaction after controlling for demographic variables, functional status, and non-motor symptoms. In this study, lower income was associated with depression, but this association disappeared when resilience was added to the regression model (24). Fatigue, suicidal ideation, and lack of resilience were all found to be significantly associated with the severity of depression in both patients and their caregivers (25). A study of patients with Lewy body disorders, including 55 patients with PD (15 with mild cognitive impairment and 40 with dementia) found an association between lower resilience and both lower quality of life and higher frequency of neuropsychiatric symptoms, including depression (26). A study of the impact of the stress of the first phase of the COVID-19 pandemic on PD patients found that the score on the Beck Depression Inventory (BID) increased during this period, but patients with higher resilience maintained lower BDI scores (27). An inverse association between resilience and depression scores was also found in 50 PD patients soon after the COVID-19 pandemic restrictions were lifted (28).

To sum up, studies that examined the relationship between resilience and depression in PD found associations of resilience with optimism and better quality of life and with less apathy, depression, fatigue, and suicidal ideation (23–28). *These studies, however, did not assess demoralization*, and it is conceivable that demoralized patients were misclassified as “depressed.” This cross-sectional observational study aimed at filling this knowledge gap by examining the interrelations of demoralization, depression, and resilience in the same group of patients with PD and the implications of those interrelations. The objective of this research was to determine if higher resilience in patients with PD is associated with lower demoralization, lower depression, or both.

## Method

### Study design, setting, and participants

This was an observational study with a cross-sectional design. Outpatients with PD were recruited from the Movement Disorders Clinic (of A.S.P.) at Yale-New Haven Hospital, a private tertiary healthcare system. Inclusion criteria were English comprehension/literacy and age 40–90 years. Exclusion criteria were current use of recreational drugs, history of suicidal ideation, diagnosis of neurocognitive disorder (dementia), and terminal illness. Age 40 was the lower limit because younger patients may experience PD differently due to their unique life circumstances (29). Use of recreational drugs was an exclusion criterion because it may, at times, manifest itself as a movement disorder complicating the diagnosis and interpretation of the findings (30). Patients with suicidal ideation were excluded because we intended to study them separately. Patients with a history of neurocognitive disorder (dementia) were excluded because resilience interventions in patients with neurocognitive disorder do not appear to have a significant benefit on depression and neuropsychiatric behavioral symptoms (31). Every attempt was made to avoid a self-selection bias by obtaining as high a participation rate as possible. A total of 133 eligible patients were invited to participate. Of these, 38 declined (not interested) giving a participation rate of 71.4%. Those who declined were similar to the participants in age and sex distributions but more likely to have more severe PD, i.e., Hoehn and Yahr stage III or IV (39.5% vs. 11.5%, *p* = 0.0002) (32).

### Variables, data sources, and assessments

A movement disorders neurologist (A.S.P.) diagnosed PD using United Kingdom Brain Bank Society criteria, assigned the Hoehn and Yahr stage, assessed for dyskinesia and evaluated for motor function using the Movement Disorders Society Sponsored Revision of the Unified PD Rating Scale, Part III (MDS-UPDRS-m) (32–36). Scored from the history given by patients and caregivers and a medical examination, Hoehn and Yahr scale distinguishes five severity stages based on the extent of involvement and functional impairment (32). MDS-UPDRS-m Part III assesses impaired motor function on a 5-point scale (none to severe) (35, 36).

Trained research assistants administered questionnaires in person after clinic appointments. Age, sex, race-ethnicity, marital status, recreational drug use, history of suicidal ideation or attempt, years since PD diagnosis, and treatment with deep brain stimulation, antiparkinsonian medications, and levodopa were self-reported and validated by chart review. Level of education, household size, and family income were self-reported. Other diseases and smoking and drinking habits were reported with a standard systems review form validated by chart review.

Demoralization was assessed with the Demoralization Scale (DS); depression, with the Patient Health Questionnaire-9 (PHQ-9); resilience, with the Brief Resilience Scale (BRS) (37–39). DS has 24 items, rated from 0 (never) to 4 (all the time) and the total score is the sum of the scores on 5 subscales (loss of meaning and purpose, dysphoria, disheartenment, helplessness, and sense of failure), with higher scores indicating higher demoralization (37). PHQ-9 has 9 items based on the DSM-IV criteria for major depressive disorder and scores ranging from “0” (“not at all”) to “3” (nearly every day) (38). BRS has 6 questions (3 positively and 3 negatively worded) (39). All scales have adequate reliability and validity and have been widely used in research, including research on PD.

### Statistical analysis

#### Sampling

With a prevalence of demoralization of 18.1% among outpatients with PD, to reach a margin error of 8%, at a confidence level of 95%, at least 89 participants would be required. To reach a power of 0.8, a sample size of at least 55 is required to detect a medium effect size (*f*^2^ = 0.15) in multiple linear regression with 1 predictor at a two-tailed α level of 0.05 (40).

#### Bivariable analyzes

Using the recommended cut-off points, the scores on the DS (37, 41), PHQ-9 (38), and BRS (39) scales were categorized as follows: low demoralization (LDem) (DS ≤ 19), and moderate to high demoralization (MHDem) (DS > 19); low depression (LDep) (PHQ ≤ 9), and moderate to high depression (MHDep) (PHQ > 9); low (LR) (BRS < 3), normal (NR) (BRS 3–4.30), and high resilience (HR) (BRS > 4.30). The bivariable correlations between demoralization, depression, and resilience were examined using Spearman rho correlation (*r*_s_) for continuous variables and chi-square tests for categorical variables. One-way ANOVA, chi-square test, Kruskal Wallistest were used to examine the bivariable relationship between resilience level and other measured variables, as appropriate (42).

#### Multivariable analysis

Because opinions differ about demoralization being continuous or categorical, separate analyzes were conducted under each assumption. Two linear regressions identified the unique associations of resilience (predictor) with depression (using demoralization as covariable) and with demoralization (using depression as covariable), treating the three variables as continuous. Omitted (unmeasured) variables may create spurious relationships among variables and attenuate causal relationships. To address this omitted variable bias (spuriousness), the regression analyzes were enhanced with covariable adjustment and copula correction. (a) *Covariable adjustment:* Age, dyskinesia, hypertension, and MDS-UPDRS score were included as control variables because they showed significant bivariable associations with demoralization and/or depression in our previous studies (4–7). (b) *Copula correction to the unadjusted regressions:* Bias in the estimates of causal effects was reduced by modifying each regression for a copula term that specified the dependence structure between the endogenous variable (resilience) and the error, with no extra covariables required. This method has proved robust to address the spuriousness caused by unmeasured confounders when the regressors are non-normally distributed (43). In our data, resilience (predictor variable) had moderate to large skewness (−0.87).

Categorical interrelations of demoralization, depression, and resilience were examined with correspondence analysis. A row and column profile analysis of the contingency table exposes the interrelations both within and between groups of variables and allows a graphical representation (44). While chi-square tests show only that a relationship exists, correspondence analysis shows how the variables are interrelated (45). The correlations between column categories (demoralization-depression severity) and row categories (resilience levels) were visually interpreted following the instructions given by Kim (46): the strength of the connection between two categories is determined by the angle formed by the lines drawn from the origin (0,0) to the categories (i.e., the correlation would be close to +1 if the angle were close to zero; close to zero if the angle were close to 90°; and close to −1 if the angle were close to 180°).

The copula correction term was calculated and models were estimated using REndo package in R (47). All other statistical analyzes were performed using SPSS v 23 (48). The quality of data collection was monitored regularly for accuracy and completeness. For all tests performed, the significance level was set *a priori* at 0.05 (two-tailed).

## Results

### Description of the sample

The 95 participants (participation rate = 71.4%) were mostly male (66.3%), white (91.6%), married (70.5%), with a college degree or higher (70.5%). Age range was 44–84 years (mean = 67.1 years, SD = 8.39 years). Only two (2.1%) were cigarette smokers; 34 (35.8%) drank alcoholic beverages socially; most were in Hoehn and Yahr stages I or II (87.4%), and none had a history of mental disorders or psychiatric treatment. Median time since PD diagnosis was 6 years (IQR = 7 years). Dyskinesia was noted in 21 (22.1%). All but 4 were treated with antiparkinsonian medications (95.8%); 60 (84.2%) with L-dopa; and 17 (22.1%) with deep brain stimulation. The 17 participants who received deep brain stimulation did not differ in demoralization or depression from the remaining participants. There were no dropouts.

### Bivariable analysis

The bivariable correlations between demoralization, depression, and resilience were statistically significant. In particular, resilience was inversely correlated with demoralization (*r*_s_ = −0.57, *p* < 0.001) and depression (*r*_s_ = −0.48, *p* < 0.001), whereas demoralization and depression were positively correlated with each other (*r*_s_ = 0.59, *p* < 0.001). The bivariable correlations between resilience and variables other than depression and demoralization were not statistically significant (Table 1).

**Table 1:** Correlates of resilience.

Variables	Resilience		
	Low (BRS < 3) *n* = 11	Normal (BRS 3–4.3) *n* = 43	High (BRS > 4.3) *n* = 41
Age [mean ± SD]	64.2 ± 9.9	68.9 ± 8.4	67.7 ± 7.8
Sex: Male [*N* (%)]	5 (45.5%)	27 (62.8%)	31 (75.6%)
Race-ethnicity [*N* (%)]			
White (Caucasian)	10 (90.9%)	40 (93.0%)	37 (90.2%)
Other	1 (9.1%)	3 (7.0%)	4 (9.8%)
Marital status, *n* (%)			
Never married	2 (18.2%)	1 (2.3%)	2 (4.9%)
Married	6 (54.6%)	29 (67.4%)	32 (78%)
Separated/Divorced	2 (18.2%)	9 (21.0%)	5 (12.2%)
Widowed	1 (9.1%)	4 (9.3%)	2 (4.9%)
Education, *n* (%)			
College or higher	9 (81.8%)	31 (72.1%)	27 (65.9%)
Secondary or Primary	2 (18.2%)	12 (27.9%)	14 (34.1%)
Currently employed, *n* (%)	4 (36.4%)	10 (23.3%)	15 (36.6%)
Income level, *n* (%)			
High (>$6,000/month)	3 (42.9%)	17 (45.9%)	17 (51.5%)
Middle ($3,001-6,000/month)	1 (14.3%)	11 (29.7%)	10 (30.3%)
Low (<$3,001/month)	3 (42.9%)	9 (24.3%)	6 (18.2%)
Cigarette smoking	0 (0%)	1 (2.4%)	1 (2.6%)
Drinking alcoholic beverages	4 (40%)	19 (46.3%)	11 (28.2%)
Dyskinesia	4 (36.4%)	8 (19.5%)	9 (22.5%)
Hypertension	3 (27.3%)	21 (48.8%)	21 (51.2%)
Motor function (MDS-UPDRS-Part III)	22.7 ± 10.06	26.0 ± 12.61	24.2 ± 11.45
Hoehn and Yahr stage, *n* (%)			
I	1(9.1%)	8(19.5%)	13(31.7%)
II	9(81.8%)	28(68.3%)	24(58.5%)
III	1(9.1%)	3(7.3%)	4(9.8%)
IV	0(0.0%)	2(4.9%)	0(0.0%)
Treatment with anti-Parkinson medications, *n* (%)	11(100%)	42(97.7%)	38(92.7%)
Currently on levo-dopa, *n* (%)	11(100%)	37(86.0%)	32(78.0%)
Treatment with deep brain stimulation (DBS), *n* (%)	3(27.3%)	6(14.0%)	8(19.5%)
Years since PD diagnosis, median (IQR)	6(7)	6(8)	6(8.5)
Mean depression score (PHQ-9) (*)	12.5 ± 6.5	6.2 ± 4.7	3.6 ± 3.3
Mean demoralization score (DS) (**)	32.2 ± 20.7	11.8 ± 12.4	5.2 ± 5.3

*F = 17.96, *p* < 0.001; **F = 24.43, *p* < 0.001.

### Multivariable analyzes

Table 2 shows the results of multiple regressions of resilience on demoralization and depression for three types of models: the unadjusted one (model 1); the covariable-adjusted one (model 2); and the copula-corrected one (model 3). The analyzes yielded consistent results across the three models, indicating that demoralization explains the association between resilience and depression. Resilience makes a unique and protective contribution to demoralization while having a non-significant association with depression.

**Table 2:** Results of multiple regression analysis.

Variables		Model 1^b^		Model 2^c^		Model 3^d^	
Predictor	Outcome^a^	β (95% CI)	SE	β (95% CI)	SE	β (95% CI)	SE
Resilience	Demoralization	−7.32 (−9.86 to −4.79)***	1.28	−7.35 (−9.74 to −4.95)***	1.20	−10.56 (−15.21 to −4.62)***	2.67
	Depression	−0.85 (−2.10 to 0.40)	0.63	−0.64 (−1.92 to 0.63)	0.64	−0.33 (−2.50 to 1.80)	1.08
	Depression (PHQ-9 > 9)	OR (95% CI)	SE	OR (95% CI)	SE	OR (95% CI)	SE
		0.56 (0.22 to 1.43)	0.48	0.48 (0.17 to 1.42)	0.55	-	-

CI, confidence interval; SE, standard error; OR, odds ratio.

^a^Demoralization is defined as the DS score; Depression is defined as the PHQ-9 score. Adjusted for depression with demoralization as the outcome; Adjusted for demoralization with depression as the outcome. ^c^Model 1 additionally adjusted for age, dyskinesia, hypertension, and MDS-UPDRS score. ^d^Copula correction to model 1. ***p < 0.001.

**Figure 1:**
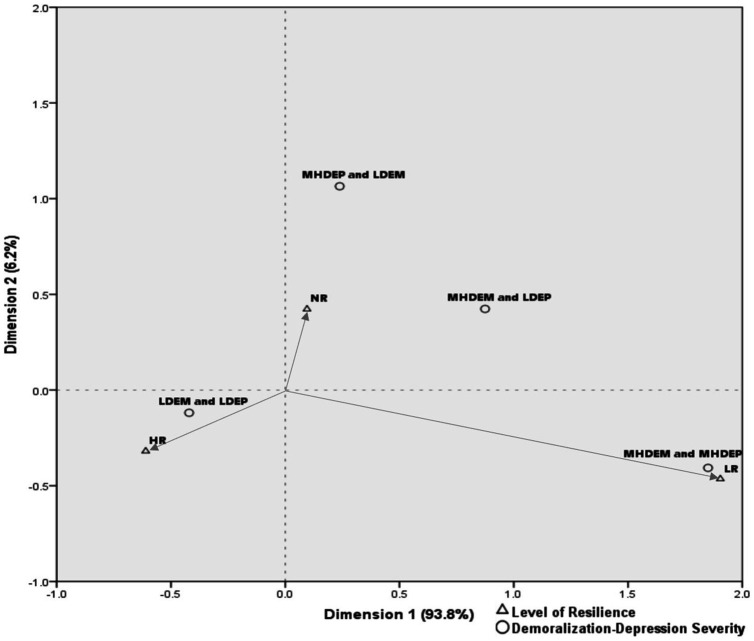
Biplot of correspondence analysis for demoralization-depression severity and levels of resilience. LDem, low demoralization; MHDem, moderate to high demoralization; MHDep, moderate to high depression; LR, low resilience; NR, normal resilience; HR, high resilience.

The correspondence analysis biplot accounted for 100% of the variance in the data. This analysis revealed participants with depression without demoralization (i.e., MHDep and LDem) to be more likely to have normal resilience; those with both demoralization and depression (i.e., MHDem and MHDep), more likely to have low resilience; and those with neither depression nor demoralization (i.e., LDep and LDem), more likely to have high resilience (Table 2; Figure 1).

## Discussion

In this cross-sectional observational study, PD outpatients were assessed for demoralization, depression, and resilience. Demographic, clinical, and treatment-related variables were also examined. The association between resilience and depression was found to be statistically significant when demoralization was not taken into consideration, but ceased to be significant when demoralization was taken into consideration. By contrast, the association between resilience and demoralization was significant when depression was not taken into consideration and continued to be significant when depression was taken into consideration. Furthermore, previous studies have shown that lower resilience is associated with greater depression (23–28), but our findings show that the impact of resilience on protecting against depression is influenced by the presence of demoralization, so that in people with lower demoralization, the role of resilience in mitigating depression becomes less important.

A case could be made that this study achieved the goal of separating depression from demoralization only partially because of the following features of PHQ-9: (a) PHQ-9 has an item (#2) that asks the respondent if he/she has been” feeling down, depressed, or hopeless” and hopelessness is a manifestation of demoralization, not depression; (b) PHQ-9 does not probe for a reduced magnitude of motivation, a characteristic of depression unshared by demoralization; and (c) PHQ-9 has an item (#9) that asks if the respondent had “thoughts that he/she would be better off dead or of hurting himself/herself in some way,” and such thoughts may occur with depression and also with demoralization (though more likely to occur with demoralization according to some studies) (16, 17). These limitations inherent to PHQ-9, however, should not have affected our conclusions because they would have biased the results in favor of depression by increasing the number of demoralized participants misclassified as “depressed.” In fact, all analyzes were repeated after deleting those two items from PHQ-9 and the same results were obtained.

These results expand our understanding of resilience by suggesting that it is a mechanism that favors reduction of demoralization over reduction of depression. The results are consistent with the hypothesis that the future orientation of resilience is based on the construction of a cognitive map to deal with perceived stress in the long run. This, in turn, appears to suggest that demoralization is a manifestation of a process originating in the cerebral cortex, and not in the sub-cortical regions of the brain. Resilience appears to involve a cerebral mechanism adapted to prevent and reduce demoralization by protecting the integrity, efficiency, and relevance of the cognitive map (direction of motivation) as long as the magnitude of motivation is relatively intact.

As Kissane noted, the appropriate intervention for demoralization is the “selection of a range of cognitively informed, existentially oriented, and meaning-centered psychotherapies” (49). If resilience reduces demoralization, such selection would likely strengthen resilience and possibly prevent hopelessness and suicide, thereby improving the prognosis of PD. A reduction of demoralization will likely be helpful to PD patients, particularly those with low resilience. Therapeutic interventions have been developed specifically tailored to reduce demoralization by modifying the perception of stress, restoring hope, and replacing negative cognitive distortions of self and stressful situations with positive and more precise and realistic appraisals. Examples are meaning-centered psychotherapy, sequential combination of cognitive-behavioral psychotherapy and well-being psychotherapy, psilocybin-assisted psychotherapy, and supportive psychotherapy at the bedside (50–54). Successful interventions to reduce demoralization and strengthen resilience are likely to improve the prognosis of PD.

The limitations of this study should be recognized. This was a cross-sectional study with a one-time assessment and no follow-up observations. Participants were outpatients at a single academic hospital, thereby limiting generalizations to patients in similar centers. The study sample consisted mainly of people with mild to moderate disability, older, white, male, married, with a college degree; results might have been different with a more diverse sample. Cross-sectional design precludes etiological inferences. Iatrogenic effects of medications used to treat PD might be unmeasured confounders (55). For example, levodopa (l-DOPA) and dopaminergic agonists have been shown to reduce (56), to have no effect (57), or to worsen the depression in PD patients (58). Data on the number of participants on dopaminergic agents were not obtained. Mean levodopa dose and its relationship to resilience and demoralization were not assessed. Positive scores on the scales employed are not the same as clinician diagnoses. Ideally, assessments should have included both clinician-rated and self-reported measures (59), but at the time of this study, there were no clinician-rated versions of the scales employed. Dyskinesia was assessed only by its presence on examination with MDS-UPDRS Part 3. Participants may not have had dyskinesia at the time of the exam but could be experiencing it at other times.

The study also has strengths. Participants were evaluated and diagnosed by a movement disorders neurologist (A.S.P.). Variables were assessed with reliable and valid scales, widely used in research, including research on PD. Statistical methods showing how the variables are interrelated and avoiding spuriousness reduced the bias in the statistical analysis.

## Conclusion

Previous studies of resilience in PD found that depression correlated with lower resilience. *These studies did not assess demoralization*. In this study of outpatients with PD, protection by resilience favored demoralization over depression. The results invite a re-examination of the role of resilience in PD patients with demoralization and expand our understanding of resilience by suggesting that it is a type of cerebral information processing mechanism evolved and adapted to prevent and reduce demoralization, not just depression. Further observational studies with longer follow-up periods are warranted to ascertain the role of resilience and demoralization in PD patients. Future research should examine resources likely to increase resilience, such as optimism, active coping, and perceived social support; identify the precise mechanisms by which resilience prevents and reduces demoralization and protects the integrity, efficiency, and relevance of the cognitive map needed to deal with the predicament of PD; and assess the efficacy of other interventions in reducing demoralization, such as mindfulness-based and acceptance and commitment psychotherapies. Preventing and reducing demoralization and strengthening resilience as part of a comprehensive treatment plan are likely to improve the prognosis of PD.

## Data availability statement

The raw data supporting the conclusions of this article will be made available by the authors, without undue reservation.

## Ethics statement

The studies involving human participants were reviewed and approved by Institutional Review Board, Yale University School of Medicine. The patients/participants provided their written informed consent to participate in this study. Written informed consent was obtained from the individual(s) for the publication of any potentially identifiable images or data included in this article.

## Author contributions

JF was the primary investigator and author, wrote the entire manuscript, and contributions included developing the hypothesis, reviewing the literature, formulating the research protocol, formulating the statistical analysis, interpreting the findings, and writing and editing the manuscript. BZ contributed with formulating and performing the statistical analyzes, interpreting the data output, helping with writing and editing the manuscript, and making the tables and the figure. RK contributed with building the database and inputting the data, and helping with developing the hypothesis, analyzing the data, interpreting the findings, and editing the manuscript. AP contributed with participant recruitment, screening potential participants, performing the neurological evaluation and assessments, and helping with interpreting the findings and editing the manuscript. BK contributed with supervising the data collection and helping with interpreting the findings, and editing the manuscript. EL contributed with facilitating the implementation of the project and helping with interpreting the findings, and editing the manuscript. All authors contributed to the article and approved the submitted version.

## Conflict of interest

The authors declare that the research was conducted in the absence of any commercial or financial relationships that could be construed as a potential conflict of interest.

## Publisher’s note

All claims expressed in this article are solely those of the authors and do not necessarily represent those of their affiliated organizations, or those of the publisher, the editors and the reviewers. Any product that may be evaluated in this article, or claim that may be made by its manufacturer, is not guaranteed or endorsed by the publisher.
